# Population Difference in Allele Frequency of HLA-C*05 and Its Correlation with COVID-19 Mortality

**DOI:** 10.3390/v12111333

**Published:** 2020-11-20

**Authors:** Atsushi Sakuraba, Haider Haider, Toshiro Sato

**Affiliations:** 1 Section of Gastroenterology, Hepatology, and Nutrition, University of Chicago Medicine, Chicago, IL 60637, USA; haiderqazan@yahoo.com; 2 Department of Organoid Medicine, Keio University School of Medicine, Tokyo 160-8582, Japan; t.sato@keio.jp; 3 Corona Virus Task Force, Keio University School of Medicine, Tokyo 160-8582, Japan

**Keywords:** COVID-19, mortality, HLA, gene, global, innate immunity, selection

## Abstract

Background: coronavirus disease 2019 (COVID-19) causes severe illness including cytokine storms, but mortality among countries differs largely. In the present study, we investigated the association between human leukocyte antigen (HLA) class I, which plays a major role in susceptibility to viral infections, and the mortality of COVID-19. Methods: data of allele frequencies of HLA-A, -B and -C and COVID-19 mortality were obtained for 74 countries from the Allele Frequency Net Database and worldometer.info. Association between allele frequency of each HLA and mortality was assessed by linear regression followed by multivariable regression. Subsequently, association of HLA-C*05 to its receptor *KIR2DS4fl*, expressed on natural killer (NK) cells, and differential mortality to historic pandemics were analyzed. Results: HLA-A*01, -B*07, -B*08, -B*44 and -C*05 were significantly associated with the risk of deaths (adjusted *p* = 0.040, 0.00081, 0.047, 0.0022, 0.00032, respectively), but only HLA-C*05 remained statistically significant (*p* = 0.000027) after multivariable regression. A 1% increase in the allele frequency of HLA-C*05 was associated with an increase of 44 deaths/million. Countries with different mortality could be categorized by the distribution of HLA-C*05 and its receptor *KIR2DS4fl*, which in combination cause NK cell-induced hyperactive immune response. Countries with similar ethnic and/or geographic background responded in a similar pattern to each pandemic. Conclusions: we demonstrated that allele frequency of HLA-C*05 and the distribution pattern with its receptor *KIR2DS4fl* strongly correlated with COVID-19 mortality. Host genetic variance of innate immunity may contribute to the difference in mortality among various countries and further investigation using patient samples is warranted.

## 1. Introduction

The symptoms of coronavirus disease 2019 (COVID-19), which is the acute respiratory syndrome caused by the virus, and severe acute respiratory syndrome coronavirus 2 (SARS-CoV-2), vary from asymptomatic to critically ill [1]. It has recently come to attention that the number of infections and deaths among countries worldwide differ largely. In China, where COVID-19 was initially identified, the infection and deaths are 58 and 3 per million population, respectively. Most Asian countries show rates in the range similar to China. In many European countries, these numbers are much greater; e.g., the United Kingdom (UK), Italy and Spain, have rates in the range of 3000–6000 and 300–800, respectively. The difference in the infection rate can be partly explained by demographic background, healthcare and governmental policies, and approach to diagnostic testing and containment of infection. However, host factors contributing to the difference in mortality due to COVID-19 remain largely unknown.

Elderly people are known to be more prone to the risks of COVID-19. The case fatality risk was reported to be 1.38% for all cases, but was substantially greater in patients ≥60 as compared to <60 (6.4% vs. 0.32%) [2]. Other risk factors include male sex, smoking, diabetes and obesity. In the United States of America (USA) and the UK, minority ethnicity groups had a higher incidence of deaths [3]. While structural inequalities contribute to difference in deaths between races in these countries, host genetic differences in susceptibility to, especially severe, COVID-19 among populations worldwide likely exist.

A majority of patients admitted for severe COVID-19 experience complications such as thromboembolism, multiorgan failure and acute respiratory distress syndrome (ARDS). Elevated levels of inflammatory cytokines such as interleukin (IL)-2R, macrophage inflammatory protein and IL-6, characteristic of cytokine storm syndrome have been found in these patients [4,5,6]. This has rationalized the use of immunomodulatory agents, tocilizumab and dexamethasone, in severe or critically ill COVID-19 patients [4,7].

Major histocompatibility complex (MHC) class I molecules are found on the cell surface of all nucleated cells. The main function of MHC class I molecules are to display intracellular peptide fragments to cytotoxic T cells and natural killer (NK) cells, which in turn induce an immune response [8]. Virus-infected host cells utilize this mechanism to trigger immune surveillance and eliminate infected cells, but the immune system can cause autoimmunity or overproduction of cytokines when regulation is impaired [9]. In humans, there are three MHC class I genes, namely human leukocyte antigen (HLA)-A, HLA-B and HLA-C. HLA loci are known as a leading genetic candidate for infectious disease susceptibility. Studies have shown that patients with certain HLA alleles are at risk for viral infections including SARS [10]. The frequency and composition of HLA among different human populations vary largely which is explained by diversifying selection, balancing selection and pathogen-driven selection [11,12]. In the present study, we investigated the association between the allele frequency of class I MHC genes, HLA-A, -B and -C, obtained from the Allele Frequency Net Database and the worldwide mortality due to COVID-19.

## 2. Materials and Methods

The data of allele frequencies of HLA-A, -B and -C genes among countries were obtained from the Allele Frequency Net Database (http://allelefrequencies.net) [13] and for mortality (deaths per one million population) were obtained from Worldometer (https://www.worldometers.info). Worldometer collects data from individual countries’ communication channels and provides global COVID-19 live statistics. It has been widely used in literature [14]. Both databases were searched on 28 May 2020. For the purpose of this study, we limited the search at the allele group level (first field). There were 79 countries with data of allele frequency of at least one HLA-A, B or C. When multiple datapoints of an allele frequency were available for a country, the weighted mean was calculated according to the sample size. Data for death/1 million population due to COVID-19 were available for 213 countries. The final dataset included 74 countries with data available for both HLA and deaths.

The analysis of the association between the allele frequency of class I MHC genes and the mortality due to COVID-19 demonstrated that HLA-C*05:01 had the strongest association. Literature has demonstrated that peptides bound to HLA-C*05:01 are recognized by an activating killer cell immunoglobulin-like receptor (KIR), KIR2DS4 [8,15]. Thus, we studied the association between the carrier frequency of the functional form of KIR2DS4, *KIR2DS4fl* and HLA-C*05. Data of frequency of carriers of *KIR2DS4fl* were obtained from the Allele Frequency Net Database [13] and also by a search of the published literature [16,17,18,19,20].

Based on the results of HLA-C*05/KIR2DS4 association, we compared the mortality due to COVID-19 among countries to those of historic global pandemics, the 1918–1920 Spanish flu and 2009 swine flu [21,22], to examine whether there is a pattern suggestive of pathogen-derived epistatic balancing selection [23].

### Statistical Analysis

The association between the allele frequency of each HLA gene and mortality was assessed by linear regression. Multiplicity adjusted *p*-values following Bonferroni multiple comparison testing was done. An adjusted *p*-value of <0.05 was considered statistically significant. Multivariate regression with genes with a *p*-value of <0.05 was performed. We then applied backward elimination with a threshold of 0.05 for selection of the most predictive variable(s). Differences were considered to be statistically significant when the *p*-value was <0.05. Data were analyzed by EZR (Saitama Medical Center, Jichi Medical University, Saitama, Japan) [24], which is a graphical user interface for R (The R Foundation for Statistical Computing, version 2.13.0, Vienna, Austria), and GraphPad Prism version 6 (San Diego, CA, USA).

## 3. Results

Data of 21, 34 and 13 HLA-A, -B and -C allele frequencies, respectively, as well as cases of death due to COVID-19 were available for 74 countries (Figure S1). Countries were distributed evenly among continents. There were 10, 25, 25, 6 and 8 countries from Africa, Asia/Oceania, Europe, North/Central America and South America, respectively.

### 3.1. Association between Worldwide Allele Frequency of HLAs and Mortality to COVID-19

**Figure 1:**
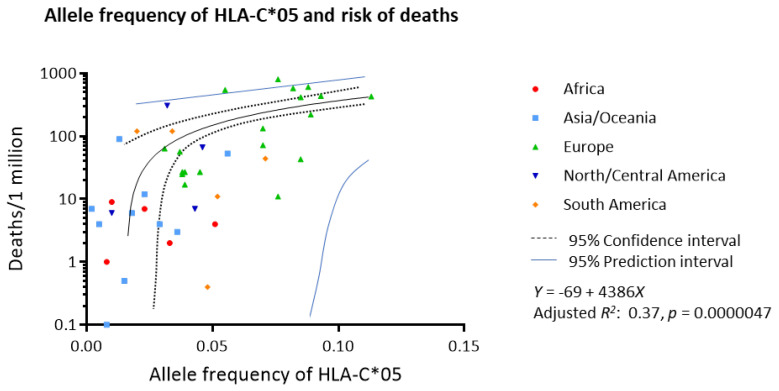
Worldwide allele frequency of HLA-C*05 and risk of deaths due to COVID-19. Linear regression showed a strong correlation between the allele frequency of HLA-C*05 and number of deaths per 1 million population (adjusted *R*^2^ 0.37, *p* = 0.0000047).

The association of each allele frequency and number of deaths due to COVID-19 per one million population was analyzed by linear regression for all HLA alleles (Table 1). After adjusting for multiplicity, HLA-A*01, -B*07, -B*08, -B*44 and -C*05 were significantly associated with the risk of deaths (adjusted *p* = 0.040, 0.00081, 0.047, 0.0022, 0.00032, respectively). Multivariate regression with backward elimination demonstrated that HLA-C*05 was the only variable that was significantly associated with the risk of deaths (*p* = 0.000027). Figure 1 shows the linear regression model of allele frequency of HLA-C*05 and the risk of deaths per million population (adjusted R^2^ 0.37, *p* = 0.0000047). It is estimated that for each 1% increase in allele frequency of HLA-C*05, mortality increased by 44 deaths per 1 million population.

### 3.2. Association between HLA-C*05 and Its Receptor, KIR2DS4

**Figure 2:**
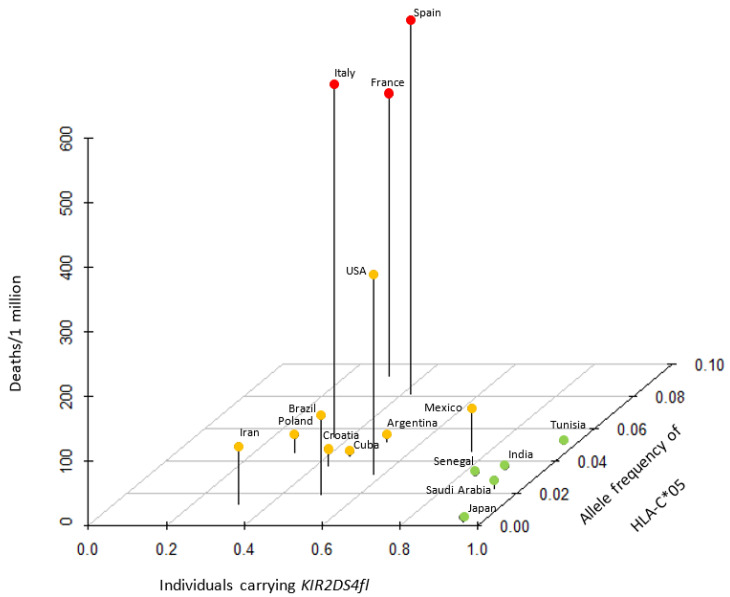
Association between allele frequency of HLA-C*05, proportion of individuals carrying *KIR2DS4fl* and risk of deaths due to COVID-19. Countries with high, medium and low mortality could be categorized into three distinct groups with a specific pattern of HLA-C*05/*KIR2DS4fl* expression. KIR: killer cell immunoglobulin-like receptor.

A recent report found that peptides bound to HLA-C*05:01 are recognized by one of the activating KIR, KIR2DS4 [8]. There are two major alleles in KIR2DS4, the full-length receptor, KIR2DS4-fl, and KIR2DS4-del that harbors a 22-bp deletion and loses membrane-anchoring [25]. KIR2DS4-fl serves as a potent activating KIR, while KIR2DS4-del is devoid of NK cell activation potential [8]. Intriguingly, KIR2DS4-del is highly associated with HLA-C*05, and both alleles are common in European populations [8], suggesting that the effective NK cell activation by these ligand-receptor pairs drove the epistasis evolution [8]. We obtained data of the frequency of carriers of *KIR2DS4fl* among 16 countries and plotted them against the allele frequency of HLA-C*05 and deaths per 1 million population (Figure 2). Countries could be separated into HLA-C*05 high/*KIR2DS4fl* medium countries that exhibited high mortality (France, Italy and Spain), HLA-C*05 medium/*KIR2DS4fl* medium countries that demonstrated medium mortality (Argentina, Brazil, Croatia, Cuba, Iran, Poland and USA) and HLA-C*05 low/*KIR2DS4fl* high countries that demonstrated low mortality (India, Japan, Mexico, Saudi Arabia, Senegal and Tunisia).

### 3.3. Distinct Pattern of Response to Historic and COVID-19 Pandemics

**Figure 3:**
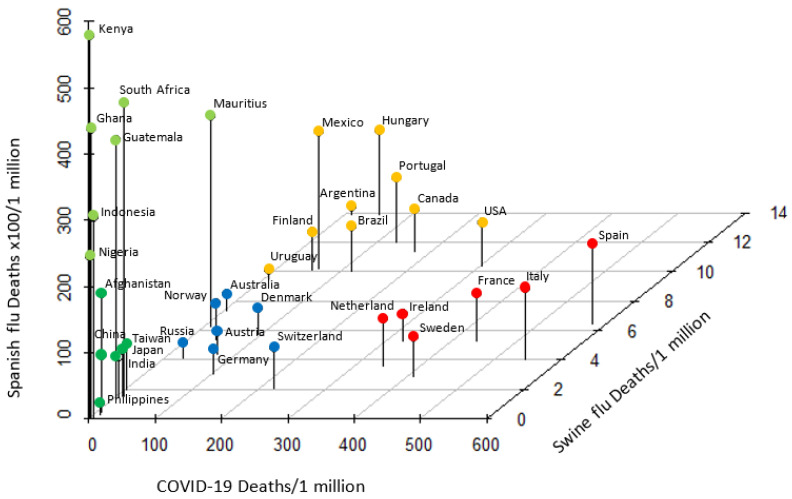
Pattern of mortality to Spanish flu, swine flu and COVID-19 pandemics. Countries with high, medium and low mortality from each pandemic were categorized into five distinct groups. With the exception of a few outliers, African, American, Asian and European countries were clustered, demonstrating a similar pattern of mortality to each pandemic.

Based upon the notion that historic pandemics have posed evolutional selection pressure on HLA alleles [26], we next compared the mortality due to COVID-19 to those of two major historic pandemics, the 1918–1920 Spanish flu and 2009 swine flu [21]. With the exception of a few outliers, countries with similar ethnic and/or geographic background showed similar degree of mortality to each pandemic (Figure 3). European countries had medium mortality to Spanish flu and swine flu, but were categorized into two groups based on COVID-19 high (Spain, Italy, France, etc.) and medium (Germany, Austria, Switzerland, etc.) mortality. Most countries in the American continent (USA, Canada, Mexico, Brazil, etc.) were clustered as Spanish flu medium/COVID-19 medium/swine flu high mortality. African countries (Kenya, Ghana, Nigeria, etc.) were categorized as Spanish flu high/COVID-19 low/swine flu low. A majority of Asian countries (Japan, Philippines, China, etc.) were clustered as low mortality to all three pandemics. Noteworthy, categorization of countries was near identical to that of HLA-C*05/*KIR2DS4fl*. Countries with similar ethnic and/or geographic background responded in a similar pattern to each global pandemic indicative of evolutionary epistatic and balancing selection precipitated by these pandemics.

## 4. Discussion

In the present study, we investigated whether a host genetic factor is associated with the risk of death due to COVID-19. Using the allele frequency data of HLA-A, -B and -C in 74 countries, we have identified five HLAs, namely HLA-A*01, -B*07, -B*08, -B*44 and -C*05, as potential candidates that increase mortality. Amongst them, HLA-C*05 was the most influential in increasing the risk of death due to COVID-19. Our results suggest that the distribution of certain HLAs at the population level account for the disparate susceptibility and mortality to COVID-19 across the world.

The HLA system harbors genes that are crucial in modulating the immune system against pathogens. HLA alleles or haplotypes are related with susceptibility to infections caused by various pathogens [10]. The frequency of HLA alleles varies among different populations or races and has been reported to influence the susceptibility to viral respiratory infections. SARS coronavirus infection, which caused an outbreak in 2003, was extremely contagious and had a high rate of mortality [27]. Taiwanese studies demonstrated associations of the HLA-B*46:01 allele with severity and the HLA-C*15:02 and -DRB1*03:01 alleles with resistance against SARS [10], however, this was not reproduced in another cohort [28]. Keicho et al. showed that the HLA-DRB1*12:02 allele was found at a higher incidence in SARS patients than in controls [29]. Furthermore, the HLA phenotype has been shown to control immune reactivity against the influenza virus [30]. Wang et al. showed that HLA-C*07:29 and B*15:2 were found more frequently in Chinese COVID-19 patients than in controls, however, their study included only 99 patients [31].

Our study has identified HLA-C*05 as the allele most strongly correlating with the risk of deaths due to COVID-19 at a global level. HLA-C molecules load nonamer peptides and function as ligands for the KIR [32]. KIR are expressed on natural killer (NK) cells and T-cells, and classified as activating or inhibitory type by the presence of inhibitory signal domain in their cytoplasmic tail [33,34]. Sim et al. reported that KIR2DS4, expressed on NK cells, detects a conserved bacterial epitope presented by HLA-C*05:01 and exerts a potent activation signal [8]. KIR2DS4 are known to recognize HLA-C-bound viral peptides, so KIR2DS4^+^ NK cells might also recognize viral peptides presented by HLA-C*05 [8]. The inverse correlation between the frequency of HLA-C*05 and the *KIR2DS4fl* alleles raised a hypothesis that this HLA-KIR pair induces immune overactivation and subsequently causes negative selection. We found that populations with the highest mortalities (France, Italy and Spain) frequently carried HLA-C*05 and *KIR2DS4fl*, while countries with lower mortality had HLA-C*05:01 low-medium/*KIR2DS4fl* high. Thus, patients with a HLA-C*05 and *KIR2DS4fl* pair, which is most frequently seen in high mortality countries, may be prone to an excess cytokine response and suffer from hypercytokinemia, characteristic of elderly patients with severe COVID-19. Of note, COVID-19 is associated with Kawasaki disease like multisystem inflammatory syndrome in children in Europe and USA, suggesting that hypercytokinemia is a hallmark of severe COVID-19 infection regardless of age [35]. Cytokine storm and HLA–KIR interaction have also been observed with severe influenza virus infections [36] and with Spanish flu which was most fatal in young adults [37]. Furthermore, by analyzing mortality to historic pandemics, we found that countries have distinct patterns of response to each pandemic. Meanwhile, HLA–KIR interaction and their selection through human evolution has been associated with the risk of preeclampsia and reproductive outcome [38,39]. These results suggested that population-level HLA-KIR diversity undergoes epistasis and pathogen-mediated selection and strongly influences the mortality to pandemics.

Limitations of our study include that we used database, but not patient-level samples for assessment of HLA. The Allele Frequency Net database has been widely used to compare immune gene frequencies among different countries, but they may not accurately represent the population of a country. The spread and casualty of SARS-CoV-2 within a country can be assessed by several methods, but we chose deaths as our outcome as it is thought that most countries would universally test a hospitalized patient with interstitial pneumonitis to rule out COVID-19. However, testing criteria for COVID-19 vary and there may be underreporting especially in hard-hit countries during the peak of infection. Furthermore, there may be other factors that may influence the spread or outcome of COVID-19 among countries, such as mask-compliance rates, lockdown policies, healthcare provisions and ICU beds per capita. We are aware that our analysis is preliminary as the COVID-19 pandemic is still progressing and the infection may not have fully penetrated in some countries. Furthermore, there is a risk for second and third waves as we have seen in past viral pandemics and according to observance of countries/areas that lifted lockdowns. While our results suggest the presence of a correlation between HLA-C*05 and death due to COVID-19, prospective genetic studies will be required to study causal relationships [40]. During the preparation of this paper, the first genome-wide association study (GWAS) study of severe COVID-19 with respiratory failure was reported [41]. This study failed to show significant allele association of classical HLA loci, however, they only used genome data from Spanish and Italian populations and used blood donors with unknown COVID-19 status as controls, which may have precluded the detection of weaker correlations among classical HLA loci. On the other hand, a preprint study of Chinese patients with COVID-19 reported that TMEM189-UBE2V1, involved in the IL-1 signaling pathway, and the HLA-A*11:01, -B*51:01 and -C*14:02 alleles were significantly associated with severe disease or worse outcome [42]. This suggests that there may be other risk and/or protective HLA(s) or HLA haplotypes among different races.

Ever since the beginning of the COVID-19 pandemic, researchers and the community have been perplexed by the large variation in the fatality seen among countries. We studied the association between class I MHC, HLA-A, -B and -C, and the risk of deaths due to COVID-19 at a worldwide level and have identified HLA-C*05 as a potential candidate that may influence mortality. The results of our study demonstrated a correlation, so additional research of the functional properties of HLA-C*05 and a subsequent study to assess causal relationship are warranted. If our results are upheld, then the situation will represent a substantial opportunity for modifying the strategy to protect against or treat COVID-19 amongst countries with varying degree of risks.

## Figures and Tables

**Table 1:** Results of linear regression and adjusted *p* values. HLA: human leukocyte antigen.

HLA	Adjusted *R*^2^	*p* Value	Adjusted *p* Value	HLA	Adjusted *R*^2^	*p* Value	Adjusted *p* Value	HLA	Adjusted *R*^2^	*p* Value	Adjusted *p* Value
A*01	0.16	0.00059	0.040 *	B*07	0.24	0.000012	0.00081 *	C*01	−0.0016	0.34	1
A*02	0.017	0.15	1	B*08	0.16	0.00070	0.047 *	C*02	−0.013	0.51	1
A*03	0.10	0.0046	0.31	B*13	0.042	0.057	1	C*03	−0.024	0.86	1
A*11	0.020	0.13	1	B*14	0.15	0.0015	0.099	C*04	−0.012	0.49	1
A*23	0.032	0.090	1	B*15	0.026	0.10	1	C*05	0.37	0.0000047	0.00032 *
A*24	0.032	0.078	1	B*18	−0.011	0.59	1	C*06	−0.019	0.63	1
A*25	0.022	0.15	1	B*27	−0.0061	0.44	1	C*07	0.18	0.0023	0.15
A*26	−0.013	0.69	1	B*35	−0.0094	0.55	1	C*08	−0.00044	0.33	1
A*29	0.066	0.021	1	B*37	−0.014	0.67	1	C*12	−0.022	0.84	1
A*30	0.0081	0.22	1	B*38	−0.0057	0.42	1	C*15	0.064	0.051	1
A*31	0.012	0.58	1	B*39	−0.0079	0.48	1	C*16	−0.029	0.90	1
A*32	0.014	0.18	1	B*40	0.030	0.096	1	C*17	0.021	0.19	1
A*33	0.11	0.0038	0.26	B*41	−0.0027	0.36	1	C*18	0.11	0.09	1
A*34	0.030	0.12	1	B*42	0.057	0.054	1				
A*36	0.011	0.23	1	B*44	0.22	0.000032	0.0022 *				
A*43	−0.022	0.53	1	B*45	0.027	0.12	1				
A*66	0.041	0.078	1	B*46	0.042	0.12	1				
A*68	−0.0094	0.53	1	B*47	0.012	0.22	1				
A*69	−0.0073	0.42	1	B*48	0.0049	0.27	1				
A*74	0.051	0.048	1	B*49	−0.018	0.86	1				
A*80	0.020	0.20	1	B*50	−0.0062	0.43	1				
				B*51	0.012	0.18	1				
				B*52	0.072	0.020	1				
				B*53	0.030	0.10	1				
				B*54	0.010	0.26	1				
				B*55	−0.016	0.82	1				
				B*56	0.020	0.16	1				
				B*57	0.033	0.076	1				
				B*58	0.091	0.0089	0.60				
				B*59	0.060	0.19	1				
				B*67	0.053	0.14	1				
				B*73	−0.029	0.83	1				
				B*81	0.021	0.24	1				
				B*82	0.030	0.28	1				

* indicates *p* < 0.05.

## Supplementary Materials

The following are available online at https://www.mdpi.com/1999-4915/12/11/1333/s1, Figure S1: Allele frequencies of class I HLAs among countries in each continent.
