# Supplementary figures and images for: Population Difference in Allele Frequency of HLA-C*05 and Its Correlation with COVID-19 Mortality
Source: Viruses. 2020 Nov 20;12(11):1333. doi: 10.3390/v12111333 (PMC7699862; doi:10.3390/v12111333)

Supplementary Figure 1. Allele frequencies of class I HLAs among countries in each continent

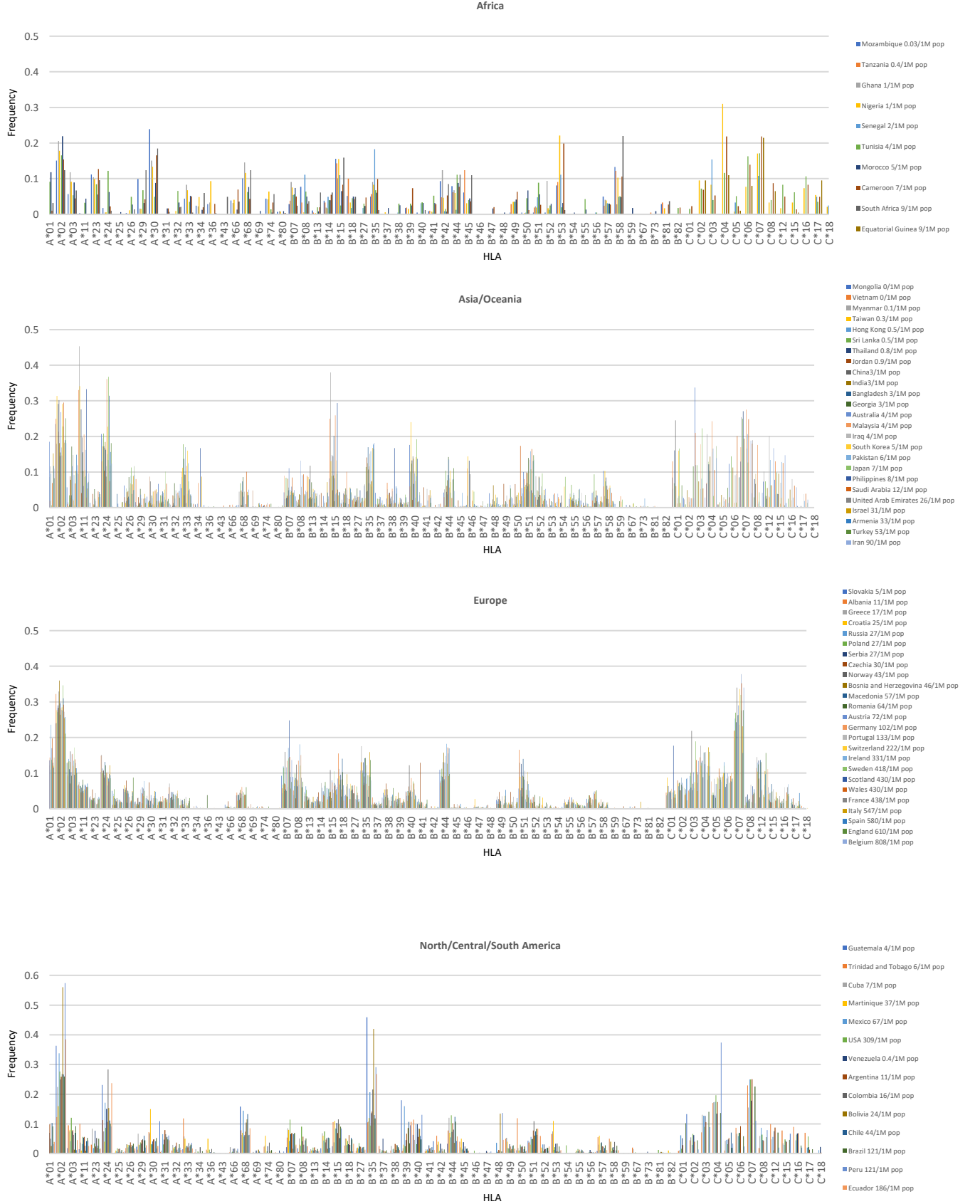

Supplement: Supplementary file 1 [file viruses-12-01333-s001.pdf]
